# Molecular Markers for Interspecies Transmission of Avian Influenza Viruses in Mammalian Hosts

**DOI:** 10.3390/ijms18122706

**Published:** 2017-12-13

**Authors:** Khristine Kaith S. Lloren, Taehyung Lee, Jin Jung Kwon, Min-Suk Song

**Affiliations:** 1 College of Medicine and Medical Research Institute, Chungbuk National University, Chungdae-ro 1, Seowon-Ku, Cheongju 28644, Korea; kaithlloren@gmail.com (K.K.S.L.); pigf22@naver.com (T.L.); lvsspacechild@chungbuk.ac.kr (J.J.K.); 2 College of Veterinary Medicine and Agricultural Sciences, De La Salle Araneta University, 303 Victoneta Avenue, Potrero Malabon City 1475, Philippines

**Keywords:** avian influenza, interspecies transmission, mammalian host, molecular marker

## Abstract

In the last decade, a wide range of avian influenza viruses (AIVs) have infected various mammalian hosts and continuously threaten both human and animal health. It is a result of overcoming the inter-species barrier which is mostly associated with gene reassortment and accumulation of mutations in their gene segments. Several recent studies have shed insights into the phenotypic and genetic changes that are involved in the interspecies transmission of AIVs. These studies have a major focus on transmission from avian to mammalian species due to the high zoonotic potential of the viruses. As more mammalian species have been infected with these viruses, there is higher risk of genetic evolution of these viruses that may lead to the next human pandemic which represents and raises public health concern. Thus, understanding the mechanism of interspecies transmission and molecular determinants through which the emerging AIVs can acquire the ability to transmit to humans and other mammals is an important key in evaluating the potential risk caused by AIVs among humans. Here, we summarize previous and recent studies on molecular markers that are specifically involved in the transmission of avian-derived influenza viruses to various mammalian hosts including humans, pigs, horses, dogs, and marine mammals.

## 1. Introduction

The influenza A virus (IAV) is an enveloped, segmented, negative sense, single-stranded RNA virus of the Orthomyxoviridae family that can be classified into different subtypes based on the genetic and antigenic properties of the two surface glycoproteins, hemagglutinin (HA) and neuraminidase (NA). The influenza A nomenclature is based on the combination of specific HA (H1–H18) and NA (N1–N11) subtypes, which theoretically generates 198 subtypes. Wild waterfowls or aquatic birds are the natural reservoir hosts of all influenza A subtypes [1] except for the two novel IAV subtypes H17N10 and H18N11, which were recently identified in bats [2,3]. With avian species as the original hosts of these mammalian IAVs with typical sub-clinical infection in cases of low pathogenic avian influenza (LPAI), AIVs can also infect a wide range of host species, including humans [4], pigs [5], horses [6], dogs [7], cats [8], and marine mammals [9].

Interspecies transmission that occurs from wild birds, such as aquatic birds of the order *Anseriformes* (ducks, geese, swans) and *Charadriiformes* (gulls, terns, waders), to terrestrial poultry (chickens, turkeys, quails, etc.) is more frequently reported in most surveillance works than that from birds to mammals [10]. Even though domestic birds (chickens, turkeys, quails, etc.) of the order *Galliformes* are not natural reservoir hosts of AIV, they are susceptible to infection with wild-bird derived IAVs and act as an intermediate host of the virus that infects humans [11]. Initially, influenza viruses were introduced from wild birds, in which subtypes H5, H6, H7 and H9 have become prevalent in terrestrial poultry especially in domestic chickens, to domestic birds [10]. Among the poultry species, turkey was probably the most susceptible with more than 10 subtypes of wild bird-origin influenza viruses detected in this bird, but none of these subtypes became established or were prevalent for a long time [10]. Few molecular features have been identified in the transmission and host adaptation of the virus to domestic gallinaceous poultry, which include mutations in both HA and NA [12,13] and viral RNP proteins [14], maintenance of HA receptor binding specificity for α2,3-SA [15] and deletion of approximately 20 amino acids in the NA stalk region [16].

In early years, it was originally believed that AIVs could not be easily transmitted directly from birds to humans due to their strong receptor binding specificity in avian species, but in 1997, the first human infection case of a highly pathogenic avian influenza (HPAI) virus H5N1 in Hong Kong was confirmed to be the result of direct transmission from poultry to humans [17]. Relatively, the host-specific influenza viruses that are circulating in other mammalian species, including dogs [7,18] and horses [19,20] are also thought to have been directly derived from avian influenza viruses. Aside from avian and terrestrial species, marine mammals (e.g., whales and seals) have also received some virologic attention, but corresponding researches are limited due to poor accessibility. Nevertheless, these few studies suggest that influenza infection in marine mammals occurs sporadically from avian sources [21].

As an enzootic virus in wild migratory birds, the original reservoir of IAVs, this virus occasionally spills over directly or via an intermediate host into other animal hosts, including humans. Although the virus usually causes a dead-end infection, it may also lead to adaptation, sustained transmission and establishment in the new host. Some subtypes such as H1N1, H2N2 and H3N2 caused previous pandemics, with genes of avian decent, have become established in the human population, and some of them are responsible for seasonal outbreaks every year [11,22]. In horse species, two subtypes, H3N8 and H7N7, are known to have become established [6,23]. Also, previous phylogenetic studies of these viruses suggest that all these equine lineages were derived from AIVs [24]. Additionally, avian-origin H1N1 lineage has been established in European pigs since 1979 [5,25]. Some avian influenza viruses can also spill-over such as the HPAI H5N1 which was detected in captive animals including tigers and leopards [26,27,28] and civets [29] that were thought to have eaten infected wild birds or were fed infected poultry carcasses. Thus, phylogenetic studies suggest that all mammalian IAV strains ultimately derive from the avian IAV pool [15].

With respect to the molecular basis of influenza virus transmission, there are many contributing factors associated with the ability of the virus to breach the avian-human host species barrier. The molecular traits of influenza virus that have been identified as important for interspecies transmission are: (i) HA receptor binding specificity; (ii) HA fusion stability; (iii) addition or deletion of a potential *N*-glycosylation site in HA; (iv) increased viral replication by RNP complex in a new host; (v) HA and NA balance in a new host [30,31]; and (vi) gene constellation by genetic reassortments [32].

The viral proteins hemagglutinin (HA) and the polymerase complex (PB2, PB1 and PA) are well studied and are specifically identified to have a vital role in viral transmission in relation to their distinct function. HA functions as the receptor binding glycoprotein that binds to cell-surface sialylated glycoproteins. Particularly, influenza viruses predominantly bind to α2,3-linked sialic acid (SA) receptors abundantly located in the lower respiratory tract of birds and the intestinal tract with respect to avian influenza viruses, to α2,6-linked SA receptors which are predominant in the human upper respiratory tract with respect to human influenza viruses, and to both linkages (α2,3 and α2,6-linked SA receptors) with respect to swine influenza viruses. This makes receptor binding of influenza viruses species-specific [33]. The previous pandemic viruses were analyzed for their receptor recognition and some of them were found to have amino-acid substitutions (e.g., Q226L, H3 numbering throughout the manuscript) in the HA receptor-binding domain (RBD) which resulted due to a change in the receptor binding preference from α2,3-linked SA receptors in avian viruses to α2,6-linked SA receptors in human viruses and serve as the prerequisite for cross-species transmission [34]. Aside from substitutions in the HA RBD, deletion of a *N*-linked glycosylation site located near the RBD at position 158–160 was found to be favorable for human receptor binding affinity and was also found to play a role in airborne transmission of H5N1 viruses [35,36,37,38]. Consequently, upon virus attachment, a conformational change in the HA protein is required for the fusion reaction between the viral and endosomal membranes, after which viral ribonucleoproteins are released in the cytoplasm of the infected cell. The fusion reaction is triggered by low endosomal pH, thus, some studies also suggest that the stability of HA in an acidic environment is another factor involved in the interspecies transmission of avian influenza viruses [36,39].

The viral RNA polymerase was also found to promote interspecies transmission of avian influenza viruses. Particularly, the adaptation of influenza virus polymerase complex (PB2, PB1, PA) in a mammalian host has been shown to be crucial for interspecies transmission due to its important role in vRNA transcription and replication and a major determinant of virus pathogenicity and host adaptation [36,37,40,41,42,43,44]. Since the RNA polymerases (PB2, PB1, PA) in avian influenza virus have relatively poor activity in mammalian cells, these polymerases, particularly basic polymerase PB2, can acquire a variety of adaptive substitutions, as the virus moves from one host to another, which contribute to host-range restriction and are important for virus adaptation in mammalian species. The high virus replication in the upper respiratory tract, as mediated by the polymerases, is critical for airborne transmission of avian influenza virus in mammals [38]. Of the internal viral proteins associated with RNA polymerase activity, PB2 was the first polymerase protein in which a mutation that helped overcome host restriction was identified [45]. Some mutations in PB1, namely at positions 473V and 598P, have also been found to increase the polymerase activity of avian influenza virus in mammalian cells [46,47]. Currently, studies assessing the possible role of other viral genes such as non-structural protein 1 (NS1) and nuclear export protein (NEP) in mammalian transmission are being performed [48,49].

Since the genome of IAV is composed of eight segments, it allows reassortment between different strains, and this ability allows the virus to constantly reinvent itself and pose a threat to human and animal health. Three out of the four human influenza pandemics were the result of reassortment of genes of avian, human or swine influenza virus origin [50]. Extensive studies on pandemic H1N1 2009 have identified the gene segments that contribute to the transmissibility. Multiple reassortment events between different avian influenza viruses have also led to increased replication and transmission in mammals. Some known subtypes that have emerged from this phenomenon are the human H7N9 [51,52] and H5N1 [37,39] viruses derived from poultry, as well as H1N2 [53,54] and H3N2v [55,56] viruses derived from swine. In particular, mammalian adaptation markers harbored by these viruses were found to be associated with transmission in animal models such as guinea pigs [39,43,57,58] and ferrets [36,37,59,60,61,62]. Thus, reassortment has also been importantly involved in the evolution of AIV as well as in interspecies transmission events [11,56,63,64,65].

## 2. Avian Influenza Virus Transmission in Various Mammals

### 2.1. Molecular Determinants Potentially Related to Transmission of AIVs to Humans

Most of the reported human cases of influenza infection usually had a history of exposure to live animals or infected poultry such as in poultry farms and live poultry markets. Some avian influenza virus subtypes (H5, H6, H7, H9 and H10) are considered to represent pandemic threats as there have been reports and studies showing that they have somehow breached the interspecies barrier and infected humans. The first case of direct transmission of avian viruses from poultry to humans was documented in 1997 in Hong Kong which was the result of a large outbreak of HPAI H5N1 in poultry [4,66] that consequently spread throughout the continent. As of 27 September 2017, 860 cases of human HPAI H5N1 virus infection have been reported to the World Health Organization, of which half were fatal [67]. Fortunately, there is no direct evidence of efficient human-to-human transmission of HPAI H5N1, a requirement for the occurrence of a pandemic infection. However, because HPAI H5N1 can infect humans, it is feared that H5N1 may mutate or reassort with circulating human influenza viruses that may enable the virus to adapt to humans and can be efficiently be transmitted from human to human. With a wide diversity of AIVs, multiple reassortment events can occur between different AIVs that result in emerging viruses such as HPAI H5Nx, of which the novel reassortant avian H5N6 virus caused human infection [68,69]. Further, such events allowed the novel LPAI H7N9 virus to emerge and was detected in China in March 2013, causing a total of 1564 laboratory-confirmed human infections with at least 612 deaths as reported by the World Health Organization [22]. The clinical signs caused by this virus are only mild and subclinical in poultry, making it difficult to detect this virus, although it can cause severe pneumonia and acute respiratory distress in most of the humans. Some other reported subtypes of avian influenza viruses that have caused human infections include H9N2 with one recent human case reported in China in 2017 [67], novel LPAI H6N1 with only one and the first human case detected in Taiwan in 2013 [70,71], and LPAI H10N7 that caused infection in abattoir workers in March 2010 [72]. The occurrence of human infections with avian influenza viruses has led to further epidemiological studies as well as molecular characterization and analysis to identify distinct signatures that confer the ability to cross the species barrier. In the preceding sections, we summarized the putative molecular markers for IAVs that are associated with the transmission of avian-derived AIVs to different mammalian species in Appendix Table A1.

#### 2.1.1. H5 Subtype

Currently, HPAI H5N1 represents a major threat to both animal and human health due to its unique characteristics. Although HPAI H5N1 has been involved in hundreds of human infections, this specific subtype lacks the ability of sustained transmission in humans which may be due to the avian-type receptor-binding specificity in H5 HA [73,74] which means that most of the HPAI H5N1 viruses preferentially bind to Siaα2,3 Gal, but not to Siaα2,6 Gal [75]. However, numerous studies on human isolated H5N1 viruses have identified molecular determinants that could be associated with sporadic infection of these avian viruses in humans especially mutations in HA that affect its receptor-binding preference either by increasing the binding to Siaα2,6 Gal or decreasing the binding to Siaα2,3 Gal [73,76]. In this past few years, several amino acid changes including Q226L, G228S, R193K, and E190D in HA have been identified in naturally acquired and experimentally introduced amino acid mutations that increased the binding of HPAI H5N1 HA to α2,6-linked SA [73,74,76,77,78,79,80].

Mutations at positions 226 and 228 (Q226L and G228S) (H3 numbering) have been previously identified in avian H2 and H3 HA [74,81,82] and they changed the binding preference from avian- to human-type which has been similarly observed in experimentally introduced mutations in the H5N1 virus isolate in 1997 (A/HK/156/1997) [83]. A related study found that a similar mutation (Q226L) in H5 HA does not confer increased binding to α2,6-glycans [74] but when Q226L is in combination with G228S mutation in HA, the binding to human-type receptors is increased, but no complete switch from avian- to human-type receptor-binding specificity was observed [74].

Several studies have used animal models such as guinea pigs and ferrets to test the transmissibility of highly pathogenic avian H5N1 influenza viruses including human isolates. Herfst et al. [36] tested an HPAI H5N1 virus A/Indonesia/5/05, harboring experimentally introduced HA Q226L and -G228S and PB2-E627K mutation in ferrets, but virus transmission via respiratory droplets did not occur. However, when the virus was forced to adapt to replication in the mammalian upper respiratory tract, the virus was transmitted after ten consecutive passages in ferrets. Additionally, three other mutations (H99Y in PB1; H110Y and T160A in HA) were suggested to be essential for the respiratory droplet transmissibility of this virus among ferrets [36]; as reviewed in a study [84]. In the study by Imai et al. [37], N224K and Q226L mutations that changed the receptor binding preference yielded an airborne transmissible H5, but additional substitutions in HA (N158D and T318I) may also be required. However, this reassortant with the mutant HA together with the remaining seven segments of A/California/04/2009 failed to transmit in ferrets, but virus transmission occurred after two passages in ferrets [37].

Experimentally generated H5N1 viruses with mutations known to affect receptor-binding specificity (E190D, K193S, Q226L, and/or G228S in HA) were demonstrated by Maines et al. [73] to determine if these human-type amino acids introduced in HA of H5N1 lead to a shift in the receptor-binding preference that may confer airborne transmissibility. H5N1 mutants encoding 226L/228S or 187G/190D/193S/226L/228S in HA recognized Siaα2,6 Gal in addition to Siaα2,3 Gal and a mutant possessing R193K, Q226L and G228S demonstrated a receptor-binding shift from avian- to human-type receptor but did not transmit in the ferret model [73].

Other studies for possible transmission of H5N1 viruses to humans were conducted using guinea pigs as the animal model, which has both avian- and human-like airway influenza virus receptors [85]. The loss of the glycosylation site near the RBD at position 158–160 is beneficial for human receptor specificity as observed in H5N1 with T160A amino acid substitutions, enhancing the binding to human-type receptor [35,36,37]. Although molecular changes in the genes of H5N1 that lead to a shift in receptor-binding specificity have been identified, they are not sufficient for H5N1 virus transmissibility in mammals [86]. Neumann et al. [86] correlated the lack of the glycosylation site (HA 154–156) and the recent human H5N1 virus infections particularly the H5N1 lineage in clade 2.2 circulating in Egypt. Human H5N1 viruses isolated from Vietnam and Indonesia have the HA 154–156 glycosylation site, unlike the H5N1 viruses isolated from infected individuals in Egypt that lack the HA 154–156 glycosylation site while 28% of H5N1 viruses circulating in avian species in Egypt in 2009–2011 possess this site [86]. The phylogenetic analysis by Neumann et al. [86] suggested that the loss of the *N*-linked glycosylation site occurred in birds and the virus was subsequently transmitted to humans. Thus, this recent analysis suggested that avian H5N1 viruses lacking the glycosylation site as observed in H5N1 lineage in Egypt transmit more readily from poultry to humans than those that possess the glycosylation site [86].

Avian influenza virus isolates exclusively contain glutamic acid at position 627 in PB2 while it is frequently mutated to lysine in human-derived isolates [45]. Mutation at this site, namely PB2-E627K, was also shown to be a requirement for conferring airborne transmissibility to HPAI H5N1 in ferrets [36,60] and high viral replication in mice [87]. It was also noted that avian virus subtypes (H5N1 and H7N9) that retained the avian-like glutamic acid at position 627 of PB2 frequently cause fatal human infections [46]. However, PB2-E627K is not always strictly necessary for avian viruses to infect mammals [88]. Another mutation that was recently found to increase the polymerase activity in conjunction with PB2-E627K is PB2-K526R, which was interestingly found in all subtypes of seasonal influenza viruses, as well as in H5 and H7 human isolates, but PB2-K526R was rare in avian isolates [89]. PB2 D701N substitution also conferred aerosol transmission in guinea pigs as a substitute for the lack of PB2-E627K [43] and it was primarily found in a duck H5N1 virus that was highly pathogenic to mice [90,91]. Interestingly, the two well-studied mammalian adaptive mutations, E627K and D701N, do not seem to be simultaneously induced in the *PB2* gene of the adapted viruses [92,93]. A study on reassortment between HPAI avian H5N1 virus with genes from pandemic 2009 H1N1 virus has found that the *PA* and *NS* genes conferred respiratory droplet transmissibility to the H5N1 virus in guinea pigs and the *NA* and *M* genes of the human virus also assisted in transmission of H5N1 through respiratory droplets in this animal model [39]. Of note, an avian-origin IAV PB1 protein with amino acids 473V and 598P was found to contribute to the polymerase activity of H5N1 virus in mammalian cells, as well as to efficient replication of the pH1N1 strain when acquired with 473V in PB1 [47]. Even though no human cases of H5N8 have been reported, a genomic analysis of H5N8 that belongs to clade 2.3.4.4., as related to the human infecting H5N6, was found to exhibit some human-like signatures including PA-404S, PB2-613I and PB2-702R, and the HA protein had substitutions S137A and S227R within the receptor-binding site as well as A160T in the glycosylation site that have been suggested to potentially increase the binding preference for human-type receptors and increased transmissibility of this virus [35,94]. Although molecular markers associated with human infection by HPAI H5N6 were not fully evaluated via the experimental study, some markers previously observed in other subtype of viruses were also observed including deletion of *N*-glycosylation at 156N in HA which possibly increases α2-6-SA receptor binding preference, E627K or D701N in PB2, which enhances viral replication in mammalian hosts [95].

#### 2.1.2. H7 Subtype

A novel avian-origin IAV of the subtype H7N9 was isolated from the first human case infected with this virus in Eastern China. As of 2017, there have been five-wave epidemics of H7N9 in China and the death rate and spatial distribution of outbreak related to the management of live poultry markets was significantly different between each wave [96]. The isolated H7N9 has most likely emerged from various reassortment events among avian viruses and identified as a reassortant virus with *HA* and *NA* mostly related to the avian influenza A H7N3 isolate from a duck and the H7N9 isolate from an unidentified wild bird, respectively [84,97]. In addition, previous studies have reported that H7N9 viruses have dual avian/human receptor-binding specificity that likely explains their ability to transmit via respiratory droplets, although this virus is less efficiently transmitted than the human influenza viruses [59,62,97,98]. A Q226L substitution in HA, a human virus-type, was observed in H7N9 isolated in China in 2013, but it retained the avian-type amino acid, glycine at position 228 [99]. In addition, unlike avian H7N9 isolates, most of the human isolates possess E627K substitution in the *PB2* gene of H7N9 virus or Q591K, D701N, and M535L substitutions to potentially compensate for PB2 627K [100] and most of them are associated with enhanced polymerase activity in mammalian hosts [101,102]. Similar results on a study of E627K and D710N mutations in PB2 of H7N9 viruses have also been observed by Zhu et al. [103]. Recent epidemiologic study analyzed that E627K in PB2, R294K in NA and V100A in PA mutations were markedly associated with an increased fatality rate in humans [104]. Recently, in a study by Qi et al. [105], mutations E627K and K526R in PB2 were found in three of the four human H7N9 HPAIVs, which are also associated with mammalian adaptation similarly observed in H5N1 [89]. Although there is currently no evidence of sustained human-to-human transmission of H7N9 at the genetic level [106], the recent report of HPAI H7N9 mutants detected in chickens in China pose an increased threat to humans [107].

An HPAI H7N7 virus also infected a number of people and a large outbreak among humans who had a close contact with infected poultry was reported in Netherlands in 2003, causing conjunctivitis and/or mild influenza-like illness [108]. A single A135T (A143T in original reference) [30] substitution in HA of H7N7 virus was found to enhance the replication kinetics in MDCK cells and A135T was suggested to have introduced a potential glycosylation site at position 141, near the receptor binding site, which may affect the receptor binding specificity [30]. Of note, two substitutions, E627K in PB2 and K404R (K416R in original reference) [30] in *HA* gene of the human H7N7 virus isolate, which have been suggested to have potentially resulted from virus adaptation to the new host [30].

#### 2.1.3. H9 Subtype

H9 influenza virus infections have been sporadically reported in humans, with one recent reported case from China [67]. However, H9 influenza viruses are widely circulating in poultry and are also isolated from pigs. Several lineages of H9 viruses including G1, G9, and Y439 have been circulating in Eastern and Southern China [109,110]. Recently, 117 genotypes have been identified in H9N2 viruses isolated from avian, mammalian and human hosts in China from 1994 to 2013 and of them, G57 genotype contributed to the country-wide outbreak of H9N2 viruses and the subsequent emergence of the novel reassortant subtypes with six H9N2 internal genes [111]. Importantly, the H9N2 viruses play the critical role of a donor of their internal genes to other viruses including H5N1 [112,113,114] and H7N9 viruses [51,115,116] that have been causing severe human infections. Although the precise molecular mechanism of human infection with viruses possessing H9N2 internal genes has not been fully understood, occurrence of recombination in the *PB1* gene might be associated with human infection by the H7N9 virus [117]. L226 in the *HA* gene of H9N2 viruses caused enhanced efficiency of direct transmissibility in a ferret model [61] and it was found that increased prevalence of the L226-possessing H9N2 in China elevated the potential for human infection with the AIV subtype [118,119,120]. Relatively, the characterization of receptor-binding specificity, replication capability and transmission in mammals of naturally isolated H9N2 AIVs from 2009 to 2013 in China have been performed by Li et al. [121]. Thirty-five viruses that have been studied represented 17 genotypes and all of which were found to preferentially bind to the human-like receptor. Six of nine H9N2 viruses tested by Li et al. [121] were transmissible in ferrets by respiratory droplet of which some of these H9N2 readily acquired the 627K or 701N mutation in PB2 upon infection in ferrets.

#### 2.1.4. Other Subtypes (H6 and H10)

An H6 influenza virus was first isolated from a turkey in Massachusetts in 1965, and since then, it has been isolated from wild, aquatic and terrestrial avian species [58,122] and humans with flu-like symptoms in Taiwan in 2013, and the virus was identified as H6N1 [123]. In a related molecular study, 34% of the H6 avian influenza viruses recognized the human-type receptor although their affinity for the avian-type receptor still remained higher [57,58]. Qu et al. [57] also found a single amino acid change in the *HA* gene at position 226, from glutamine to leucine, which caused a shift in the receptor-binding preference from avian-type to human-type receptors of A/chicken/Guangdong/S1312/2010 (H6N2) and contributed to respiratory droplet transmission in guinea pigs [58]. A related recent study found that a single nucleotide substitution that resulted in amino acid change at position 225 (G225D) completely switched the binding specificity of H6N1 to human-type receptors with significant binding to human tracheal tissue and loss of binding to chicken tracheal epithelium [124].

Several H10 subtypes have caused diseases in other mammals [125,126,127], and H10N7 had caused an outbreak in abattoir workers in Australia in 2010 [72] and a recent novel reassortant of an avian-origin H10N8 has also infected humans [128,129,130], although the human-infecting H10N8 virus strongly binds to avian-type receptors [131,132]. Phylogenetic studies showed that the human H10N8 virus resulted from reassortment of H9N2 strains with other avian virus isolates and even isolates from the environment, wherein the *HA* and *NA* genes originated from ducks and wild birds [128,133,134,135]. Specifically, six internal genes (*PB2*, *PB1*, *PA*, *NP*, *M*, *NS*) of human H10N8 were derived from poultry H9N2 virus that most likely reassort with the HA from avian H10N3 and *NA* from avian H10N8 or H3N8, which was determined by the highest percentage of similarity of sequences [128,133]. The human-isolated H10N8 possessed E627K in *PB2* gene which is typically observed during mammalian adaptation of AIVs [128,133]. In addition, recent mutagenesis study found that A588V in *PB2* gene of H10N8 human isolate plays critical role in viral polymerase activity and replication in mammalian cell [136].

### 2.2. Molecular Determinants Potentially Related to Transmission of AIVs to Swine

The swine species has been previously proposed as a prime ‘mixing vessel’ or intermediate host for the reassortment of human and avian influenza viruses [1] due to its dual susceptibility to avian and human IAV strains in association with the presence of both α2,3 and α2,6 SA linkages on the pig tracheal epithelium [137]. The first mammalian influenza virus, known as the ‘classical’ swine H1N1 virus, was isolated as early as 1931 from swine in North America [138,139,140] which was likely derived from the 1918 pandemic [139]. The ‘classical’ swine H1N1 subsequently caused enzootic seasonal disease in pigs in North America for about 70 years until the triple reassortant H3N2 viruses emerged in 1998, containing *NP*, *M* and *NS* genes from classical-swine H1N1 viruses, *PB1*, *HA* and *NA* from human H3N2 viruses, and *PB2* and *PA* from avian viruses [141,142]. A number of fully avian viruses (e.g., H4N6, H3N3) have been isolated from swine in North America, but none of these viruses seem to have become established in the swine host [143,144]. In Europe, a distinct wholly avian H1N1, the Eurasian avian-like H1N1, which appeared to have very well adapted to the swine host, was detected and isolated from European swine in 1979 and it was found to be closely related to a similar virus strain isolated from wild ducks [5,25], with all of the gene segments of the prototype viruses being of avian origin [145].

The first documented isolated wholly avian influenza virus from pigs in North America was named H4N6, and it was similar to the influenza viruses found to replicate in the epithelial cells of the gastrointestinal tract of ducks [143]. An encoded L226 in the HA has shown to be a major determinant of infectivity of primary swine and human respiratory epithelial cells in some H4N6 viruses [146,147]. Another amino acid change, particularly asparagine at position 193 in the HA of a Eurasian lineage avian H4N2, was also recently shown to be critical for the binding to α-2,6-linked SA, and N193 could be a possible molecular marker for predicting the binding of avian H4 viruses to α-2,6-linked SA [148].

A wholly H3N3 avian virus was also detected in pigs in North America and it was examined for signatures related to receptor preferences [144]. The swine H3N3 isolate maintains the glutamine, typical of avian viruses, at residue 226 of the *HA* gene, but it has an alanine at residue 228 in contrast to glycine in avian viruses and serine in human viruses [144].

H9N2 viruses have also been isolated from swine and they appear to be closely related to viruses isolated from chicken [149]. A leucine at position 226 within the receptor-binding pocket, which is associated with the human receptor binding preference [81], was found in four swine H9N2 virus isolates [149]. Amino acid changes in the HA at residue 227 within the receptor-binding pocket and residues 274, 279, and 286 outside the receptor-binding area were found to be unique to the swine H9N2 isolates but their significance remains unknown [149]. In addition, position 200 (position 190 in the reference) [149] has also been reported to influence the affinity for the human receptor (α2,6 SA), with Val at this position conferring highest affinity, Thr at this position conferring intermediate affinity, and Ala at this position conferring weakest affinity [150]. Of note, two of the pig H9N2 isolates studied have Ala at position 200 (190 in the reference) [149] while still having preference for the α2,6-SA [149]. H9N2 has also previously been demonstrated to have compatibility for reassortment with other viruses (e.g., pH1N1) resulting in many reassortants that have increased infectivity, transmissibility and pathogenicity via an experimental study in animal models [61,151,152] including pigs [153].

Unlike other avian viruses that have been transmitted to other mammals, the E627K substitution in PB2, which is known to increase the replication ability of avian influenza viruses in mammalian hosts [154,155], was not found in transmission pairs of avian and swine isolates in a study by Karnbunchob et al. [156]. All of the avian-to-swine transmission pairs analyzed possessed E in both avian and swine, thus suggesting that the E627K amino acid substitution in the PB2 protein is not needed for causing infection in swine hosts by AIVs [156]. However, R340K and I478V substitutions in PB2 tend to be associated with avian viruses involved in avian-to-swine transmissions [156]. In addition, some identified amino acid positions as signature residues for avian-to-human transmission [157] have also been found in avian-to-swine transmission, which include positions 199, 588, 613, and 674 in PB2, positions 327 and 336 in PB1, and position 57 in PA [156].

### 2.3. Molecular Determinants Potentially Related to Transmission of AIVs to Equine and Canine

Equine influenza virus causes a common respiratory infection in horses and other equids around the world, wherein two subtypes, H7N7 (equine-1) and H3N8 (equine-2), have evolved from avian influenza virus ancestors [21] and have been established in horses, although H7N7 has not been isolated for several decades and is considered to be extinct from equids [6]. The influenza A (H3N8) viruses currently circulating in horses were first isolated in 1963 with prototype influenza A/equine/Miami/63 [158] and the *HA* lineage of H3 has probably diverged from an avian virus ancestor around 1952 [19]. The novel strain, A/equine/Jilin/89 virus, which appeared in horses in China, caused an outbreak with approximately 400 equine deaths, but A/equine/Jilin/89 virus was not a member of the equine-2 EIV lineage [20]. All the gene segments of the novel strain were most similar to the H3N8 influenza viruses of avian species [20]. However, there are no known molecular markers that have been previously studied and identified and which are specifically associated with the transmission of avian viruses to horses, but the receptor binding characteristics of the equine-1 (H7N7) and equine-2 (H3N8) viruses were explored in a study by Suzuki et al. [159]. In relation to the receptor-binding specificity of HA in influenza viruses, they found a difference in the amino group of the SA in the tracheal epithelial cells in horses, pigs, and humans. The horse tracheal epithelial cells, which possess the predominant moiety of α-2,3 Gal SA which is similar to that in avians but different from that in humans (SA α-2,6 Gal), have 97% *N*-glycolyl type α-2,3 Gal SA, which is lacking in chickens and in contrast to *N*-acetyl type prevalent in humans [159]. This study suggests the role of HA-sialyloligosaccharide specificity in host range restriction of some influenza viruses [159].

Recently, the emergence of avian influenza viruses in companion animals such as canines has also gained attention. In 2004, influenza viruses were isolated from dogs; an H3N8 was isolated from lung tissue of greyhounds in Florida [160] and H5N1 was isolated from dogs in Thailand [7]. The unique lineage of H3N8 isolated from dogs was found to be of equine origin, which has been established and designated as canine influenza virus (CIV) [161]. However, there are also IAVs of avian origin that have affected dogs, including H5N1 and H3N2. The H5N1 virus isolated from dogs in Thailand exhibited some possible molecular markers that may be associated with species transmission such as E627K in PB2. However, the H5N1 receptor-binding site still exhibited avian characteristics, containing Q222 and G224 in the *HA* gene [7]. Additionally, all genes of the H3N2 canine isolates in Korea were found to be closely related to an avian influenza virus origin and may have been transmitted to dogs through air-borne transmission or by ingestion of infected birds [18]. The H3N2 also primarily retained the G226 and G228 in the HA, typical of avian-origin IAVs, suggesting the preferential binding of the virus to the avian-like α2,3-linked SA receptors in canines, but H3N2 was found to have a single mutation of S159A which was suggested to be an adaptation potentially resulting in increased binding to α2,3-linked SA receptors [162]. Interestingly, the mutation Leu at position 222 observed in H3N2 CIV differed from that in conserved Tryptophan at a similar position in avian H3 IAVs, suggesting the potential role of 222L in adaptation of H3N2 in dogs [162]. Lastly, the recombinant CIV HA in the study by Pulit-Penaloza et al. [162] showed a strong binding preference for α2,3-linked, mixed α2,3/α2,6-linked SAs and relatively strong binding to *N*-glycolylneuraminic acid-containing glycans that have been previously found to be predominant in the equine tracheal epithelial lining [159].

### 2.4. Molecular Determinants Potentially Related to Transmission of AIVs to Marine Mammals

As reviewed by White [9], there have been reported spill-over events of IAVs from avians, specifically wild water-birds, to seals on different occasions. The earliest virus was isolated from a marine mammal, a South Pacific striped whale, in 1976 and the virus was identified as subtype H1N3 [163], other subtypes that have also been isolated from seals include H3N8 [164,165], H3N3, H4N6 [166], H7N7 [167], and H10N7 [127]. With a close relation to various avian influenza A H10N7 viruses, the isolated H10N7 virus from seals in Northwestern Europe exhibited mutations mostly in the *HA* gene, corresponding to amino acid changes that are not found in H10 isolated from avians [125]. Amino acids at positions 351H, 379I, and 398D in HA; 17C and 453S in PB2, and 192H in PA in seal isolates were not found to be present in any of the available Eurasian avian virus sequences used in the study by Bodewes et al. [125]. Importantly, the only genetic marker known for potential adaptation to mammals that was detected in seal influenza (H10N7) virus in Europe was 220L in HA [125,168], suggesting that the amino acid changes observed might be specific for seals and H10N7 viruses [125]. H3N8 was also isolated from harbor seals in New England in the 2011 outbreak [164]. The isolated H3N8 has naturally acquired the D701N substitution which has not been identified in previous outbreaks of influenza in seals, but it is consistently found in equine and dog H3N8 viruses, suggesting its possible association with virulence and transmission of this virus between mammals [164]. In addition, the H3N8 virus isolated from seals still maintains the avian phenotype Q226 and G228 in HA which still correlates with the SAα-2,3 binding preference [164]. Interestingly, amino acids PB2-701N and HA-260M were shared exclusively by the seal, and canine and equine H3N8 viruses, suggesting adaptation to mammalian hosts [164].

## 3. Concluding Remarks

There are several molecular features that allow the avian influenza viruses to breach the barriers to inter-species transmission and even contribute to their adaptation and replication which may lead to their establishment in a new mammalian host. In some cases, other than the amino acid changes in the genes of the viruses, the contribution of receptor specificity to host range restriction also depends on the combination of host animal binding receptor type and virus strain, just as in the case of the unique and dominant type of SA found in the trachea of horses [159]. As observed in most of the transmission studies, putative mutations, especially in the *HA* gene, mostly those that affect the receptor-binding specificity, stability, and glycosylation, play an important role in the ability of the viruses to transmit to mammals. Thus, in numerous studies, the viral HA protein has been identified as the major host restriction factor, limiting the interspecies transmission and even establishing viral lineages in new hosts, although there is evidence for the contributing role of the viral polymerase complex (PB2, PB1, PA), especially in the virulence, host range, virus adaptation and replication. However, even though all of the markers identified do not have similar effects in other influenza viruses, identification of these putative mutations in different genes and subtypes of avian viruses that are related to species transmission is important for assessing the risk that may be induced by potential or newly emerging strains and it raises public health concern. While some of the possible molecular markers or signature substitutions have resulted from virus adaptation to the new host, such as the well-known E627K, the other markers may already be present in the avian viruses prior to transmission to their new mammalian host [30]. Thus, it is necessary to continuously monitor the genetic composition of the circulating influenza strains in various species, regardless of the pathogenicity (LPAI or HPAI), as well as to assess the infection and potential for interspecies transmission. However, monitoring the genetic composition should also be supported by phenotypic assays to further assess the threat posed by transmissible avian influenza viruses. In spite of the dynamic interplay among the environment, animals, and humans, reassortment of avian viruses with contemporary mammalian influenza viruses has not been presently detected in nature, as shown by the limited number of subtypes affecting humans and other mammals. However, since numerous studies have shown the possibility of transmission of wholly avian virus or mainly avian-derived virus to various mammals as possibly determined by some molecular markers, both in natural avian influenza viruses and laboratory-generated viruses, the high level of diversity in avian influenza viruses may also provide new opportunities for reassortment that may lead to new pandemics or could establish a new lineage in a new mammalian host. Thus, control of influenza viruses, continuous global surveillance, as well as understanding the mechanism of interspecies transmission and molecular determinants through which the emerging avian influenza viruses can acquire the ability to transmit to humans and other mammals are important keys in evaluating their potential risk to public health.

## Appendix A

**Table A1:** List and characteristics of selected (putative) molecular markers for transmission of avian influenza to various mammalian hosts.

Host	Gene	Amino Acid Position ^a^	Subtypes	Specific Virus Tested	Remarks	References
Humans	*PB2*	K526R	H5, H7	A/Zhejiang/DTID-ZJU01/2013 A/chicken/Zheijang/DTID-ZJU01/2013 A/Indonesia/5/2005 A/Guangdong/ST798/2008	increased polymerase activity; enhanced virus replication	[89]
		A588V	H7N9	A/Guangdong/GH074/2013	higher polymerase activity; efficient replication in mammalian and avian cells; higher virulence in mice	[136]
			H9N2	A/Lengshuitan/11197/2013		
				A/chicken/Guangdong/V/2008		
			H10N8	A/Jiangxi-Donghu/346-1/2013		
		Q591K, D701N, M535L, T271A	H7N9	A/Shanghai/2/2013 A/Dk/JX/3286/2009	enhanced polymerase activity in mammalian host	[100,101,102]
		E627K	H5N1	A/Indonesia/5/2005	transmission in ferrets after several passages; high viral replication in mice	[36,60,87]
			H10N8	A/Jiangxi-Donghu/346-1/2013	associated with mammalian adaptation	[169]
				A/Jiangxi-Donghu/346-2/2013		
		D701N	H3N2, H5N1	A/Panama/2007/99 A/Vietnam/1203/04	aerosol transmission in guinea pigs	[43]
	*PB1*	H99Y	H5N1	A/Indonesia/5/2005	transmissibilty in ferrets	[36]
		473V; 598P	H5N1	A/Cambodia/P0322095/2005	enhanced polymerase activity in mammalian cells; efficient virus replication	[47]
	*HA*	A135T (A143T) ^b^	H7N7	A/Netherlands/33/03	enhanced replication	[30]
				A/Netherlands/219/03		
		T160A	H5N1	A/duck/Guangxi/35/2001 A/barheaded goose/Qinghai/3/2005	transmissibilty in guinea pigs; enhanced binding to human-type receptor	[35]
		A186K (A182K) ^b^	H5N1	A/Thailand/1-KAN-1/04RG	Increased virus binding to α2,6	[80]
		N186K (N182K) ^b^	H5N1	A/Indonesia/5/2005	Increased virus binding to α2,6	[80]
		D187G, E190D, K193S, Q226L, G228S	H5N1	A/Hong Kong/486/97 A/Vietnam/1203/04	increased binding to α2,6	[73]
		N224K, Q226L, N168D, T318I	H5N1	in H1N1p background (A/California.04/09)	changed receptor binding preference and transmission after several passages in ferrets	[37]
		G225D	H6N1	A/Taiwan/2/13	shift in receptor-binding preference to human-type receptor	[124]
		Q226L	H7N9	A/Anhui/1/2013 A/Shanghai/2/2013	higher affinity to human receptor	[116]
			H9N2	A/Guinea Fowl/Hong Kong/WF10/99; A/Duck/Hong Kong/Y280/97; A/Chicken/Hong Kong/SF3/99; A/Hong Kong/1073/1999	enhanced transmissibility in ferrets	[61]
			H6N2	A/chicken/Guangdong/S1312/2010	shift in receptor-binding preference to human-type receptor; transmission in guinea pigs	[57]
		Q226L, G228S	H5N1	A/Indonesia/5/2005	transmission in ferrets after several passages; α2,6 recognition	[36,73]
		K404R (K416R) ^b^	H7N7	A/Netherlands/33/03; A/Netherlands/219/03	may have resulted from virus adaptation	[30]
Swine	*PB2*	R340K; I478V	not specified	not specified	involved in avian-to-swine transmission	[156]
	*HA*	T200A(T190A) ^b^	H9N2	Sw/HK/2106/98; Sw/HK/3297/98	affinity to α2,6	[149]
		Q226L	H9N2	Sw/HK/2106/9; Sw/HK/9/98; Sw/HK/10/98; Sw/HK/3297/98	Increased binding affinity to α2,6	[149]
			H4N6	A/swine/Ontario/01911-1/99; A/swine/Missouri/A01727926/2015		[146,147]
Canine	*PB2*	E627K	H3N2	A/Dog/Thailand/KU-08/04	efficient viral replication	[7]
	*HA*	S159A	H3N2	A/canine/IL/12191/2015	potential adaptation resulting to receptor binding	[162]
		W222L	H3N2	A/canine/IL/12191/2015	potential adaptation resulting to receptor binding	[162]
Seals	*PB2*	453S	H10N7	A/Seal/Sweden/SVA0546/2014	not found in any available Eurasian avian viruses	[125]
		D701N	H3N8	A/harbor seal/Massachusetts/1/11	associated with virulence and transmission	[164]
	*PA*	192H	H10N7	A/Seal/Sweden/SVA0546/2014	not found in any available Eurasian avian viruses	[125]
	*HA*	356H (351H) ^b^, 384I (379I) ^b^, 403D (398D) ^b^	H10N7	A/Seal/Sweden/SVA0546/2014	not found in any available Eurasian avian viruses	[125]
		226L (220L) ^b^	H10N7	A/harbor seal/Denmark/14-5061-1lu/2014-07	not found in any available Eurasian avian viruses	[125]

^a^ All *HA* genes are in H3 numbering; ^b^ Original numbering as stated in the reference.
